# Parental Socialization, Delinquency during Adolescence and Adjustment in Adolescents and Adult Children

**DOI:** 10.3390/bs12110448

**Published:** 2022-11-14

**Authors:** Sonia Climent-Galarza, Marta Alcaide, Oscar F. Garcia, Fangzhou Chen, Fernando Garcia

**Affiliations:** 1 Department of Developmental and Educational Psychology, Faculty of Psychology, University of Valencia, 46010 Valencia, Spain; 2 Department of Methodology of Behavioral Sciences, Faculty of Psychology, University of Valencia, 46010 Valencia, Spain

**Keywords:** parenting styles, parental socialization, adjustment, adolescents, adults

## Abstract

Although parental socialization has an influence on child development, current research is questioning which combination of parental strictness and warmth acts as protective or risk factors, especially during adolescence when the child is more vulnerable. The sample was 2125 participants, 58.7% female, divided into four age groups: adolescents (28.57%), young adults (28.38%), middle-aged adults (23.95%), and older adults (19.11%). The families were classified into four parenting styles: neglectful, indulgent, authoritative, and authoritarian according to their warmth and strictness scores. The psychosocial adjustment was measured by children’s scores on academic/professional self-concept, self-esteem, delinquency during adolescence, and benevolence values. A MANOVA 4 × 2 × 4 was applied with parenting styles, sex, and age group as independent variables. The results showed that, for adolescents and adult children, only parenting styles characterized by warmth (i.e., indulgent, and authoritative) were found to factor against delinquency during adolescence and benefit greater academic/professional self-concept, self-esteem, and benevolence values, while parenting without warmth (i.e., authoritarian, and neglectful) were identified as risk factors. Contrary to classical research, the present findings seriously question the universal benefits of strict parenting as the only optimal strategy to protect not only against delinquency, but also to foster an adequate self and the internalization of social values.

## 1. Introduction

Research mainly from Anglo-Saxon contexts with European American families has traditionally identified firm control and maturity demands (i.e., greater strictness) combined with warm and responsive strategies (i.e., greater warmth), the so-called authoritative style, as the optimal parenting as it has been consistently related to the greater adjustment and fewer problems in children and adolescents. However, studies carried out in Anglo-Saxon contexts with ethnic minority groups such as African American and Chinese American families and in Arab countries, in addition to some recent research carried out from other cultural contexts such as Europe and South American countries, seriously questions whether the combination of parental strictness and warmth (i.e., the authoritative style) is always related to universal benefits for adjustment and fewer problems in children and adolescents. Additionally, research generally examines parental socialization during childhood and especially in adolescence, a period of greater vulnerability. However, less is known about the relation between parenting and adjustment beyond adolescence once parental socialization is ended. The present study examines the relationship between parenting and outcomes (self-esteem, academic/professional self-concept, and benevolence values delinquency during adolescence) in Spanish adolescents (during parental socialization) and adult children, including young, middle-aged, and older adults. The aim of this study is to identify which parenting style (i.e., authoritative, authoritarian, indulgent, or neglectful) is related to greater adjustment (i.e., higher self-esteem, academic/professional self-concept, and benevolence values) and less delinquency during adolescence.

### 1.1. Parental Socialization

Parental socialization refers to the process by which the adult can transmit to the young person the habits and values of the culture of origin so that the child adopts adequate functioning within the culture to which the child belongs [1,2,3]. Leaving aside cultural or demographic variations of what adopting an “adequate functioning” might mean, broadly speaking, it could be said that a child will be recognized as a competent adult if he or she does not display deviant behaviors, maintains close relationships, is economically self-sufficient and socializes his or her offspring successfully—all of them are the result of education, training, and imitation [4,5]. Therefore, socializing a child with the right psychosocial development can be a challenge [6,7]. This process comes to an end when children become adults. By then, they are expected to have learned to self-regulate themselves in front of social demands without the presence of parents and to have become responsible members of society [8,9].

It should be emphasized that the socialization of children is not an exclusive task of parents. Although the family is the main (the first) socializing agent for children [9,10,11,12], it is not the only agent through which culture and social values are transferred to future generations [13,14,15]. Other agents come into play in this process, such as the school, which is also responsible for promoting the education of the young person, peers, or the media [11,12]. However, the present study will focus on studying the impact of parents on the psychosocial development of their children due to their greater degree of influence on them [5,9,16,17].

### 1.2. The Two-Dimensional Framework and Four Parenting Styles

The influence that parents exert on their children can be examined according to the two-dimensional model of Maccoby and Martin (1983) [10], which presents two dimensions, independent of each other, from which the four parenting styles emerge. These two dimensions are warmth and strictness. The first refers to the acceptance [18], sensitivity, and affection of parents when dealing with their children, the quality of the dialogue they engage in, and the use of reasoning [1,19] when children show inappropriate behavior. Recently, this parenting dimension has been called acceptance/involvement [20,21]. On the other hand, strictness is related to parental demands placed on children to promote compliance, i.e., the degree of imposition, authority, or rigidity. This parenting dimension has also been labeled in different ways as control [17,22] or firm control [23], and more recently called strictness/imposition [8].

Four parenting styles emerge from the combination of the two main parenting dimensions [1,8,10,24,25]. The authoritative style is defined by high warmth and high strictness. The indulgent style refers to high parental warmth and low strictness. The authoritarian style refers to low warmth and high strictness. Lastly, the neglectful parenting style comprises low warmth and low strictness. This model has been used as a guide to identify the best parenting style as measured by the degree of psychological well-being, development, and personal and psychosocial adjustment of children [1,8,10,21,24]. In this model of normative parental socialization, parents transmit social norms and values to their children by acting on them contingent on their behavior. It does not include non-normative parents who are not contingent on their children’s behavior (e.g., insulting the child when he/she behaves well) as well as parental practices characterized by child abuse (psychological, physical, or even sexual) and neglect (e.g., leave the teens alone for months) [1,5,7,9,10].

### 1.3. The Optimal Parenting

Traditionally, mainly from studies conducted in the United States with middle-class European American families, the combination of strictness and warmth has been identified as the best parental socialization style. The authoritative style was defined as the parenting method capable of generating the most optimal outcomes [21,26,27,28,29]. This parenting style was found to be associated with different indicators of children’s psychological well-being, such as higher optimism, life satisfaction, self-esteem, and happiness [30]. In addition, children whose parents display this parental typology appeared to have greater coping skills in the face of adverse events and, in turn, a wider range of adaptive behaviors [21,28,31,32,33]. It has also been associated with higher academic achievement, a lower likelihood of experiencing behavioral problems, and lower substance use [21,28,34]. Much of the classical psychological literature points out that parental warmth is able to provide children with the necessary emotional support, while strictness allows for the establishment of clear expected patterns in children’s behavior [1,10,21,28,29]. Recent studies found the benefits of authoritative parenting [35,36,37,38,39].

However, these classic results on the benefits of the authoritative parenting style do not always coincide with those obtained from studies conducted in other cultural contexts. Firstly, it should not be overlooked that the vast majority of studies in favor of the authoritative typology were conducted in the United States, and generally with middle-class participants of European American origin [13,21,28,40,41]. Some studies conducted on ethnic minorities in the United States report that the authoritarian parenting style (i.e., strictness without warmth) is associated with greater assertiveness and independence in African Americans [42] and an absence of mental health problems in Arab adolescents [43].

Additionally, some recent literature about parenting points out that indulgent parenting (i.e., warmth without strictness) is related to optimal child adjustment in criteria such as family self-concept [6], less aggressiveness, and sexist prejudice [3], academic and family self-esteem [13], self-esteem and school performance [44], and greater self-concept and less maladjustment [45] compared to other parenting styles. As a result, new studies question the replicability of the results and claim that the strength of this relationship varies across samples and contexts [13,30,41,46]. So much so, that this evidence against the supposed and undeniable universality of the benefits of the authoritative style has opened an endless debate regarding the best parenting style.

### 1.4. Variations in the Optimal Parenting and Culture

One way to address the current debate and explain the different results observed in different studies is through the cultural inconsistency hypothesis [24,41]. According to this hypothesis, the same parental socialization could have different consequences depending on the cultural context where parental socialization occurs, so it is possible that for a parental style to be optimal it must match the expectations of parenting subject to the sociocultural context of origin [24,41]. For example, authoritarian parenting (i.e., strictness without warmth) is related to maladjustment in children from European American families [21,28], but to some benefits in those from African American [42] and Chinese American families [20]. In other words, a parenting style that is inconsistent with the environment will not have positive effects or may even be detrimental to the child [41]. Hence, the cultural context in which socialization takes place has an important implication in the relationship between parental style and the child’s psychosocial development [47,48].

Alternative explanations in line with the cultural inconsistency hypothesis and thus against the universality of the positive effects of the authoritative typology are in line with Bronfenbrenner’s (1986) Ecological Theory [49]—which suggests that people not only fit better but are also more satisfied in environments that display their same values and attitudes—and by the cultural constructs of collectivism and individualism and their variants of verticality and horizontality [48,50,51,52]. Collectivism refers to the perception of oneself as a part of a group, while individualism refers to the view of oneself as autonomous [24,50,53]. Regardless of how one perceives oneself in relation to the collective, one can either accept inequality within the unit (vertical) or emphasize equality by seeing all members as equals (horizontal) [14,24,48,54,55].

These two lines are not only able to offer possible explanations about variations in the outcomes of the same parenting style examined in different contexts, but they also show that the authoritative typology is not always the one that presents the best results in the psychological and social well-being of children [13,24,41,48], nor is it always associated with positive outcomes as suggest some benefits related to the authoritarian parenting [20,43] and some recent evidence about the benefits of the indulgent parenting [4,8,14].

On the one hand, some benefits related to authoritarian parenting have been found in families from US minorities, and Arab or Asian countries. For example, it has been observed that in African American families that usually lived in poor and dangerous neighborhoods with greater rates of criminality and lack of opportunities, the authoritarian parenting style might not be related to detrimental consequences as in middle-class families from the US [42,56,57]. This might be explained by the fact that in these contexts, where disobeying parental rules may lead to harmful consequences, the authoritarian style, which has recently been identified as the first of the three stages of optimal parental socialization [13], brings benefits such as protection and might be an effective method in certain contexts [1,14,41]. In this sense, children from authoritarian African American families reported the highest scores in assertiveness and independence [42]. Similarly, in Arab or Asian countries where the vertical collectivist culture predominates, discipline, imposition, and rigidity are perceived as essential in parenting strategies and their lack is seen as a sign of irresponsibility [43,55,58]. Therefore, it is possible that they are understood as a form of protection instead of intrusion [42,56,59]. Therefore, the aforementioned evidence about non-detrimental consequences or even some benefits related to authoritarian parenting (i.e., strictness without warmth) revealed that, at least in some cultural contexts, the parental warmth component within the authoritative parenting style could be unnecessary in generating optimal outcomes, although parental strictness seems to be necessary and effective [42,56,57,58].

On the other hand, Latin American and Southern European countries are characterized by a horizontal collectivist culture, where affection, acceptance, and involvement in the socialization of the child seem to be necessary for optimal parenting. Unlike hierarchical cultures, in this type of culture, the group is organized on an egalitarian basis [24,48,50,54]. Therefore, parental strictness does not seem to fit into the preconceived scheme of parental socialization in these cultures—which calls into question whether the dimension of strictness within the authoritative parental style is truly indispensable for the socialization of children in collective horizontal cultures [13,24,48]. Thus, different studies carried out in countries such as Spain, Portugal, and Brazil claim the benefits that an indulgent parenting style can offer such as self-concept and the internationalization of social values in adolescents [47,48,60,61]. Additionally, other studies also support the benefits of indulgent parenting in the psychosocial adjustment of the child as greater emotional self-esteem and less emotional maladjustment in terms of nervousness, emotional instability, and hostility [8], greater emotional and family self-concept [4], higher social, emotional and family self-esteem [14].

In summary, studies conducted with ethnic minority groups, with low socioeconomic status, and in countries and cultures other than the United States do not support the positive effect linked to the authoritative style [1,20,24,41,45]. These results suggest that parenting styles are not equally effective in all cultural contexts [24,41,42,43,56].

### 1.5. Parenting and Its Contribution to Adjustment during Adolescence and Beyond

Most studies examine the relationship between parental socialization and differences in child adjustment during childhood and adolescence, which is when parents are raising their children. The influence of the family could be especially strong for children, as a family imprint, because parental socialization occurs in a developmental time of greater plasticity. Although experts in the field of development have suggested that parental socialization has crucial implications beyond adolescence [5,9,16,17], there is little empirical evidence about the long-term effects that parental socialization may have on children [3,14,62]. There is a time when parental socialization is over: when the adolescent child reaches adult age. From that moment on, although the relationship between parents and adult children is generally maintained, parents do not perform their socializing tasks (e.g., punishing their children when they misbehave) [5,8,9]. Throughout adult development, there are also important differences between those with good adjustment and those with poor adjustment [63,64]. Differences in adjustment among adult children may also be related to the type of socialization that they received, although this point has been less examined [14,65].

Development across adolescence and adulthood is usually examined through different indicators of adjustment and maladjustment. Optimal development is based on greater adjustment and poor maladjustment [66,67,68]. Adolescent delinquency is one of the most serious forms of deviance that is associated with significant personal and social maladjustment, including problems in the school and family environments [69,70], poor self-esteem [71] and self-concept [70,72], and an inadequate internalization of social values [73]. There are significant differences in the rates of delinquent behaviors (e.g., getting into fights and stealing), between normative and adjusted adolescents, compared to maladjusted and antisocial adolescents, who have higher rates [11,66,67].

Additionally, in adolescence but also adulthood, self-esteem and self-concept, and internalization of social values are indicators of optimal development. Self-esteem evolves as time goes by as follows: levels tend to be high in childhood, dropped in adolescence, increase gradually throughout adulthood, and declined in old age [74]. The global valuation of the person (i.e., self-esteem) is based on the different relevant domains of the self (i.e., self-concept) [75,76,77,78]. Performance at school and work is beneficial for personal and social adjustment; in adolescence, it is mainly linked to school and academic tasks [68,79], whereas in adulthood it ranges from preparation for work (e.g., college or vocational training), career development and retirement [63,64]. In general, self-perception as a good student or worker (i.e., academic/professional self-concept) is one of the key elements to feel a valuable person with good qualities (i.e., self-esteem) [75,77,78]. Finally, the internalization of social values represents one of the main goals of a society, although not all adolescents and adults prioritize social values [7,9,80]. A good priority towards social values, particularly those more prosocial (e.g., benevolence values) favors good self-esteem and self-concept [7], and a lower tendency towards deviance [73]. Therefore, in family studies, a key question is which parental strategies are a protective factor for optimal development, and which strategies are a risk factor. In addition, although differences in demographic variables are not the focus of parenting studies, previous research has reported some sex- and age-related differences in self-esteem, delinquency during adolescence, and benevolence values. Females tend to have higher academic/professional self-concept and benevolence values [8,81], but lower self-esteem and delinquency during adolescence than males [24,74]. As for age-related differences, a general trend suggests that there are age-related increases in the self from adolescence through middle adulthood, and then a decreasing trend through old age [8,74], while on benevolence values, adolescents tend to report lower scores than adults [81].

### 1.6. The Present Study

This study aims to analyze which parenting style (i.e., authoritative, indulgent, authoritarian, or neglectful) is related to better adjustment in adolescent and adult children (young, middle-aged, and older adults). Since the development of children is very broad and encompasses innumerable aspects, this study will capture psychological development based on academic/professional self-concept and self-esteem, delinquency during adolescence, and benevolence values.

Problems during adolescence can seriously damage adolescent adjustment, particularly when they are clearly opposed to social values and the law (e.g., delinquency) [82,83]. Some theorists have suggested the storm and stress hypothesis [84,85], based on the assumption that problems during adolescence are normative (i.e., they succeed a majority and with a positive impact). Specifically, from traditional clinical studies, they argued that adolescence is a period of “identity crisis” [85] and “individuation” [84] associated with a certain degree of discomfort, disruptiveness, and defiance for the family, but benefits in the path to healthy adulthood because adolescents must free themselves from dependence on their parents as a requirement to become adults with their own identity and sense of self and good adjustment (e.g., personal competence and acceptance of the norms) [68,86]. However, empirical evidence from community samples of adolescents suggests that problems during adolescence have negative consequences for adult adjustment [8,67,87]. The idea of crisis or problems in adolescence also underlies current studies based on a psychosocial perspective [88,89,90,91] and neuroscience [92,93], but these problems during adolescence have a negative impact on health in adulthood.

Parents exert a decisive influence in adolescence, although their influence diminishes [66,94] at the same time as that of peers increases [95,96]. Some problems can appear in adolescence such as drug use [12,97], poor academic achievement and school misconduct [21,59], aggressive behaviors [98,99], antisocial tendency [67], and even delinquency [21,24]. However, the family is not an isolated context in which socialization occurs, as it includes others such as peers, school, and the media [12,49,66,100]. During adolescence, peer approval may be based less on adult standards and more on conformity to peer standards that sometimes deviate from social norms [31,68,101]. For example, school success may be devalued by peers and negatively associated with students’ social standing [68,79], and even adolescents with high self-concept may decrease their academic engagement [96]. Despite these extrafamilial influences, parents remain the socializing agents during adolescence [1,10,21,28,29]. However, current parenting raises serious questions about which parenting style acts as a protective or risk factor for adolescents [13,41].

Overall, to protect against deviance in adolescence, parental strictness is constantly identified as a protective factor according to classical studies from middle-class European American families [21,26,27,28,29]. Those adolescents with parents who are strict, and also warm (i.e., authoritative), benefit in terms of protection against maladjustment and problems. Even adolescents from families also characterized by strictness, but without warmth and involvement (i.e., authoritarian families), scored reasonably well on measures of obedience and conformity to the social standards; they do well in school, and they are less likely than their peers to be involved in deviant activities. On the opposite side, adolescents from indulgent families, with warm and involved parents (as those from authoritative families), but lower parental strictness, report a higher frequency of substance abuse and school misconduct and are less engaged in school [21,28]. Nevertheless, some recent evidence mostly from European and Latin American countries seems to suggest the benefits of parental warmth [19,60], while parental strictness seems to be unnecessary or even detrimental [13,102] to protect against adolescent maladjustments such as drug use [44,97] and alcohol use [12] and help aggressive adolescents [99] or with an antisocial tendency [67].

The present study examined the consequences of parenting styles once parental socialization is in the process (in adolescent children), but also when is over (in adult children). The aim is to examine parenting styles and their relationships for psychosocial development during the socialization years (through delinquency during adolescence) but also for the adjustment (through self-esteem, academic/professional self-concept, and benevolence values), in both cases among adolescents and adult children. Along with the problems in adolescence related to delinquent behaviors, which are associated with major problems of personal and social adjustment [21,24,82], the psychosocial development is examined through the global (i.e., self-esteem) and the specific component of self (i.e., self-concept), and also the internalization of benevolence values. In particular, the assessment of oneself as a person with good qualities and value (i.e., self-esteem) is associated with good personal adjustment and well-being [72,103], while academic and professional competence is represented in the academic-professional self-concept, which includes the learning (e.g., school or university) and professional environment (i.e., work) [75,76,104]. Additionally, the internalization of benevolence values is associated with empathy and prosocial behaviors [105,106]. The literature on parenting has focused primarily on examining parental influence on child adjustment during the parental socialization process (i.e., childhood and adolescence) [21,24,28,40,42,44], but fewer studies examine child adjustment beyond adolescence when parental socialization has ended [3,14,62].

According to previous literature, as the present study was conducted in a European country (Spain), the following hypothesis was tested: the parenting styles associated with greater adjustment (i.e., higher self-esteem, academic/professional self-concept, and benevolence values) and less maladjustment (i.e., lower scores in delinquency during adolescence) would be the parenting styles characterized by warmth (i.e., the indulgent and authoritative). Likewise, the parenting styles with the worst adjustment are expected to be authoritarian and neglectful, both characterized by a lack of warmth. Additionally, differences in child adjustment were analyzed as a function of parenting style, but also considering the sex and age of the children, to test for possible interaction effects of parenting style with sex and age, as well as to identify sex and age differences in adjustment criteria reported in previous studies.

## 2. Materials and Methods

### 2.1. Participants and Procedure

The sample consisted of a total of 2125 participants from Spain. Specifically, 1227 females (58.7%) and 878 males (41.3%), both adolescents and adults (*M* = 36.19 and *SD* = 20.55). This sample was divided into 4 age groups: adolescents (*n* = 607, 352 females, 58%) aged 12–18 years (*M* = 16.63 and *SD* = 1.61); young adults (*n* = 603, 352 females, 58.4%) aged 19–35 years (*M* = 23.55 and *SD* = 3.72); middle-aged adults (*n* = 509, 327 females, 64.2%) aged 36–59 years (*M* = 48.35 and *SD* = 6.38); older adults (*n* = 406, 216 women, 53.2%) aged 60 years and over (*M* = 68.99 and *SD* = 8.04). The grouping of participants into different age groups is based on different previous studies [3,8,107]. It included adolescents (when parental socialization in process), and three groups of adult children (when parental socialization is finished), divided into young, middle-aged, and older adults based on the common age classification of the studies in adulthood and old age [108]. All participants had similar socioeconomic characteristics and they were from a community sample of middle-class families.

Following the a priori power analysis [109,110,111] which is usually used in studies to calculate statistical power, a minimum sample of 1724 participants was determined necessary to achieve detection with a statistical power of 0.95 (α = 0.05; β = 0.05; 1 − β = 0.95) a low effect-size, *f* = 0.10 [112,113]. With the sample size of the present study, 2125 participants, it is possible to detect a very small effect size, *f* = 0.09 (by fixing α = 0.05; β = 0.05; 1 − β = 0.95). Sample collection was carried out by collecting responses to online questionnaires with mandatory responses hosted on the university website during the 2019–2020 and 2021–2022 academic years. Participants usually take almost 45 min to answer the online survey. All the participants in this study (a) were Spanish, as were their parents and four grandparents; (b) lived in two-parent nuclear families with a mother or primary female caregiver and a father or primary male caregiver, and (c) participated voluntarily. Adolescents were recruited from the complete list of high schools through random selection. Young adult participants were recruited in undergraduate education courses. Middle-aged participants were recruited from randomly selected middle-class neighborhoods. The procedure of selection was based on door-to-door-canvassing. The different neighborhoods were stratified by quartile of average household patrimony, and three middle-class neighborhoods were randomly selected. Older adult participants were recruited by randomly selecting from the full list of senior centers. Participants signed a declaration of consent and parental consent was required for adolescent participation. In all cases, anonymity and confidentiality of responses were assured. As data protection measures, (a) identifiers (e.g., university account) and survey data were stored in separate archives and (b) directory passwords were safeguarded, and sensitive archives were encrypted. In addition, respondents were informed about their rights and freedoms following the Helsinki procedure. Participants were made aware that participation was voluntary and that they could drop out of the procedure and no longer participate in the study if they wished to do so (for a similar procedure, see Garcia et al., 2021 [67]). The questionnaires were assessed for doubtful response patterns as implausible inconsistencies between negatively and positively worded responses [114,115,116]. About 1.00% (*n* = 22) of the participants were recognized as questionable and eliminated from the sample.

### 2.2. Measures

#### 2.2.1. Parental Socialization

Parental socialization was measured with two specific scales of the PARQ Questionnaire (Parental Acceptance Rejection/Control Questionnaire): the Warmth/Affection Scale (WAS) [117,118], and the Parental Control Scale (PCS) [117,119]. Parental warmth was assessed by the Warmth/Affection Scale (WAS) [117,118]. This scale consists of 20 items and aims to measure parental involvement and affection perceived by children (e.g., “My parents make me feel loved and needed”; “My parents show me that they love me”). For adult children, the items are the same but are written in the past tense (e.g., “My parents were really interested in what I did”; “They told me how proud they were when I behaved well”). The alpha coefficient of the WAS scale was 0.944. Parental strictness was assessed by Parental Control Scale (PCS) [117,119]. The PCS scale consists of 13 items intended to measure parental control, imposition, and strictness, also perceived by children (e.g., “My parents want to control anything I do”; “My parents tell me exactly when I have to be back home when I go out”). As with the previous scale, in the adult version of the questionnaire, the PCS scale items appear in the past tense (e.g., “My parents told me that I should do things the way they wanted me to do them”; “My parents gave me tasks and did not allow me to do anything else until I finished them”). The alpha coefficient of the PCS scale was 0.902. The adult version of the WAS scale and the PCS scale have good psychometric qualities to measure parental socialization in adult children [120,121]. Both scales use a Likert-type scale with a range from 1 “almost never true” to 4 “almost always true”. Higher scores indicate a greater perception of warmth and strictness.

The four parental socialization styles were defined following the procedure of dichotomization by the median of the warmth/affection and parental control scores, examining both variables simultaneously: authoritative style (above the median in both measures), neglectful (below the median in both measures), authoritarian (below the median in warmth/affection and above in parental control), and indulgent (above the median in warmth/affection and below in parental control) [8,14,21]. For heuristic rather than diagnostic purposes, the median dichotomization procedure (rather than using predetermined cut-off points) allows families to be classified into one of the four parenting styles according to their scores, which may be above or below the median value, providing a sample-specific categorization of families [8,21,105]. For example, families in the “neglectful” household are indeed relatively more neglectful (i.e., less warm and strict) than the other parents in the sample, although we do not know whether the families we have labeled “neglectful” would be considered “neglectful” within a different population (see Lamborn et al., 1991, p. 1053 [21]). The factor structure of the Adult Version of the Parental Acceptance/Rejection Questionnaire (which includes the two subscales used in this study: the WAS and the PCS) was confirmed by confirmatory factor analysis. Its invariance by age has been assessed, making it a valid measure for assessing parental socialization in adult children [14,120,121].

#### 2.2.2. Academic/Professional Self-Concept

Academic/professional self-concept was measured by the 6 items of the Academic/professional self-concept dimension of the Multidimensional Self-Concept Scale Form-5 (AF5) [122]. This dimension encompasses how one perceives oneself in the academic/professional environment (e.g., “I do school/professional work well”), and how one feels that one’s teachers/superiors value one’s performance (e.g., “My teachers/supervisors consider me to be a good worker”). The items are answered on a Likert scale with a range from 1 (strongly disagree) to 99 (strongly agree). High scores indicate a high academic/professional self-concept. The alpha coefficient for this dimension of this AF5 scale was 0.879. The AF5 is a widely applicated questionnaire for adolescents [8,14,105] and adults [3,6,114] in Spanish language. Several empirical studies have tested the scale’s dimensional structure with exploratory analyses [122] and also with confirmatory factor analyses [116,123,124] in different cultural contexts, such as Spain [124], Portugal [125], Brazil [75], Chile [114], the United States [126] and China [76]. Specifically, the validation of the instrument has shown its invariance by sex, age, and different languages such as Brazilian Portuguese [75], Portuguese [125], Chinese [76], and English [126] with the Spanish version. In addition, no method effects were found related to negatively worded items on the AF5 scale [114,116].

#### 2.2.3. Self-Esteem

Self-esteem was measured with the Rosenberg Self-Esteem Scale (1965) [127]. This scale is one of the most widely used measures to assess the concept of global self-esteem [30]. It consists of 10 items measuring the degree of self-acceptance and self-respect and is answered on a Likert scale with a range of 1 to 4, with 1 meaning “strongly disagree” and 4 meaning “strongly agree” (e.g., “I am generally satisfied with myself”; “I have a positive attitude towards myself”). High scores denote high self-esteem. However, 5 of the items are written in negative, so they must be counted in reverse (e.g., “I often think that I am not good at anything”; “I feel that I do not have too many things to be proud of”). The total score on self-esteem is obtained with an arithmetic mean of the scores on the 10 items. The alpha coefficient for this scale was 0.848.

#### 2.2.4. Delinquency during Adolescence

Delinquency during adolescence was measured by assessing the frequency of different behaviors such as stealing, confronting the police, or being armed [21,24], exactly, with 8 items such as “Stealing goods from supermarkets (or department stores)” or “Damage strangers’ cars”. The response pattern presents 3 alternatives: “never”, “once”, and “two or more times”. Adolescents reported their current rates, while adults (youth, middle-aged and older adults) reported their rates when they were adolescents [21,67]. Self-reported measures of delinquency during adolescence are used primarily in studies with adolescents, and in studies with adults using the same measures as with adolescents, but with the items in the past tense [21,67]. The coefficient alpha was 0.657. Different scholars support the use of self-report measures to measure delinquency, even though they are subject to fluctuations both above and below the actual level of activity. They are considered to provide information closer to reality than official police reports do [21,24].

#### 2.2.5. Benevolence Values

Benevolence was measured with the benevolence subscale of the Schwartz Values Inventory (SVI) [128]. The benevolence values were: “Loyal (loyal to my friends and my belonging group)”; “Helpful (working for the welfare of others)”; “Forgiving (forgiving to others)”; “Responsible (trustworthy)”; “Honest (sincere, authentic)”, whose response pattern is reported by a Likert scale ranging from 1 “not important in my life”, to 99 “essential in my life”. The alpha coefficient for this dimension of the SVI scale was 0.730.

### 2.3. Plan of Analysis

The statistical analysis performed comprised a full multivariate factor analysis (MANOVA), followed by univariate factor analysis (ANOVA), and finally, Bonferroni post hoc tests. First, multivariate factorial design MANOVA (4 × 2 × 4) was conducted using as dependent variables academic/professional self-concept, self-esteem, delinquency during adolescence, and benevolence, while the independent variables were parenting styles (neglectful, indulgent, authoritative, and authoritarian), sex (female vs. male), and age group (adolescent, young adults, middle-aged adults, and older adults). We will use the two demographic variables (adolescent sex and age group) as independent variables (i.e., factors), rather than as statistical covariate controls, to (a) test for any possible moderation (i.e., interaction) and (b) analyze whether well-documented effects of demographic factors on the dependent variables (i.e., children’s outcomes or criterion variables) are as expected in the literature. The factors (i.e., independent variables) in the design controlled (decrease) residual error variance and increase the multivariate Λ-test and univariate *F*-test power [129,130,131]. Second, univariate tests were applied for analysis in those sources of variation that reached the statistical level of significance in the multivariate analyses. Ultimately, Bonferroni hypothesis tests were applied to the statistically significant results found in the univariate analysis while maintaining the alpha per study at 5%.

## 3. Results

### 3.1. Distribution of Parenting Styles

Table 1 provides an overview of the distribution of the participants according to the parenting style: neglectful, indulgent, authoritative, and authoritarian. On the one hand, the indulgent parenting style, *M* = 73.60 and *SD* = 4.36, and authoritative parenting style, *M* = 72.65 and *SD* = 4.19, had higher scores in warmth than the other two parenting styles, neglectful, *M* = 56.87 and *SD* = 9.18, and authoritarian, *M* = 55.30 and *SD* = 9.65. On the other hand, the authoritative parenting, *M* = 39.85 and *SD* = 4.84, and authoritarian parenting, *M* = 41.91 and *SD* = 5.47, scored higher in strictness than the neglectful parenting, *M* = 28.32 and *SD* = 5.47, and indulgent parenting, *M* = 28.28 and *SD* = 5.37.

### 3.2. Adjustment Criteria

Results from correlation analysis between adjustment criteria are presented in Table 2. Academic/professional self-concept, self-esteem, and benevolence values were positively correlated between them. In addition, academic/professional self-concept and benevolence values were negatively associated with delinquency during adolescence (see Table 2).

### 3.3. Multivariate Analysis

The results from the multivariate analysis indicated significant differences in all main effects: parenting styles, Ʌ = 0.861, *F*(12, 5529.9) = 26.83, *p* < 0.001; sex, Ʌ = 0.913, *F*(4, 2090.0) = 49.85, *p* < 0.001; and age groups, Ʌ = 0.876, *F*(12, 5529.9) = 23.58, *p* < 0.001. The interaction effects between sex and age group were also statistically significant, Ʌ = 0.963, *F*(12, 5529.9) = 6.63, *p* < 0.001 (see Table 3). Neither the interaction effect of parenting styles by sex nor parenting styles by age reached the statistically significant level (*p* > 0.05).

### 3.4. Univariate Analyses and Post-Hoc Tests

#### 3.4.1. Parenting Styles, Delinquency during Adolescence, and Adjustment

The results of the ANOVAs showed that there are statistically significant differences between parenting styles and all development criteria: academic/professional self-concept, *F*(3, 2093) = 55.71, *p* < 0.001, self-esteem, *F*(3, 2093) = 53.95, *p* < 0.001, delinquency during adolescence, *F*(3, 2093) = 12.20, *p* < 0.001, and benevolence, *F*(3, 2093) = 44.81, *p* < 0.001 (see Table 4).

A constant pattern between parenting styles, delinquency during adolescence, and adjustment was found (see Table 4). Overall, the optimal scores were related to parenting styles based on warmth (i.e., the indulgent and the authoritative). Specifically, children from indulgent and authoritative families scored higher on academic/professional self-concept, self-esteem, and benevolence. On the other hand, both parenting styles were related to the lowest scores on delinquency during adolescence. In contrast, neglectful and authoritarian parenting styles were associated with the highest scores on delinquency during adolescence, and the lowest scores on academic/professional self-concept, self-esteem, and benevolence (the latter especially for neglectful parenting).

#### 3.4.2. Sex and Age Groups

Univariate analyses also showed that there were statistically significant sex and age group differences for all the adjustment criteria measured (*p* < 0.001). Additionally, the interaction effect between sex and age reached the statistically significant level (*p* < 0.05) only for academic/professional self-concept, *F*(3, 2093) = 13.79, *p* < 0.001, and delinquency during adolescence, *F*(3, 2093) = 5.32, *p* = 0.001.

Examining sex-related differences, females had the highest scores in academic/professional self-concept and benevolence. In contrast, males had the highest scores in self-esteem and delinquency during adolescence (see Table 5).

Regarding age-related differences (see Table 6), middle-aged participants (36–59 years old) showed the highest academic/professional self-concept, followed by young adults (19–35 years old) and older adults (60 years old and above). Those with the lowest scores on this criterion were adolescents (12–18 years old). In self-esteem, young adults, middle-aged and older adults had the highest scores on this adjustment criterion, while adolescents, again, scored the lowest. Regarding delinquency during adolescence, young adults scored the highest, followed by adolescents and middle-aged adults, with older participants scoring the lowest. Finally, regarding benevolence values, middle-aged participants showed the highest search for the well-being of others, followed by adolescents and young adults.

**Figure 1:**
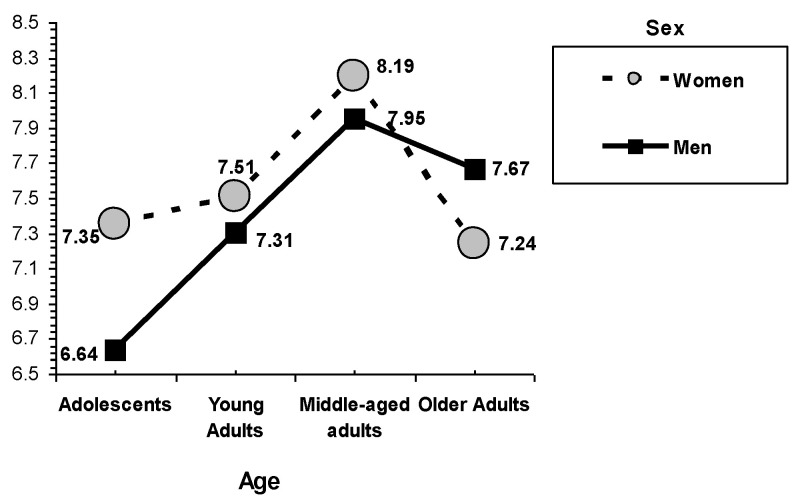
Interactions of sex and age. Academic/professional self-concept.

In the interaction between sex and age group on academic/professional self-concept (see Figure 1), it can be observed that, in general, academic/professional self-concept tends to be higher in females than in males (this tendency is noticed especially in adolescents). Adolescents had the lowest academic/professional self-concept, in particular male adolescents. However, these sex differences tended to even out as adolescence progresses. It is also noted that academic self-concept tended to increase with age (this tendency is more pronounced in males). In fact, the highest point in relation to academic/professional self-concept for both sexes is in middle age. However, academic/professional was lower among older adults compared to middle-aged adults (this pattern is more pronounced in women).

**Figure 2:**
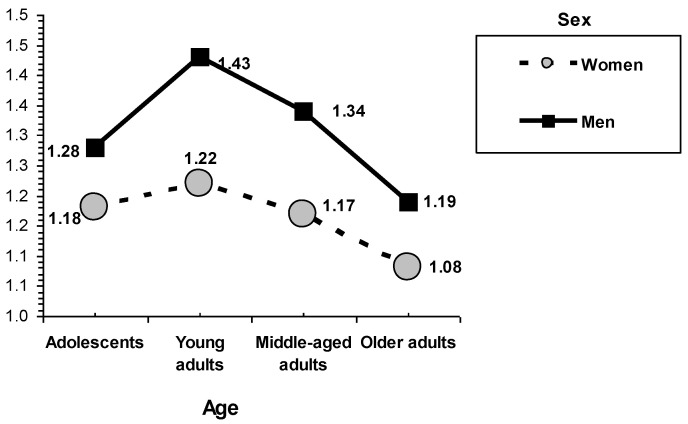
Interactions of sex and age. Delinquency during adolescence.

In the interaction between sex and age group on delinquency during adolescence (see Figure 2), it is generally found that females tend to have lower scores than males. The greater scores were reported by young adults, especially among males. The lowest scores in delinquency during adolescence were reported by older adults for both males and females. Adolescents and middle-aged adults had similar scores, lower than adolescents (a tendency especially remarkably for males), but higher than older adults (see Figure 2).

## 4. Discussion

### 4.1. Main Findings

This study analyzed the relationship between parenting styles (e.g., neglectful, indulgent, authoritative, and authoritarian), delinquency during adolescence, and adjustment based on academic/professional self-concept, self-esteem, and benevolence values. The results of the present study in a European country (Spain) revealed that children from indulgent (warmth without strictness) and authoritative (warmth with strictness) families, both adolescents and adults, are those who showed a greater psychological adjustment based on a higher academic/professional self-concept, higher self-esteem, greater benevolence values and, in turn, a lower rate of delinquency during adolescence. The lower expression of delinquent behavior points to the protective role of these two parental styles against deviance to the social standards. On the opposite side, the neglectful (neither warmth nor strictness) and authoritarian (strictness, but not warmth) styles were associated with the greatest delinquency during adolescence and the lowest adjustment in terms of academic/professional self-concept, self-esteem, and benevolence values.

### 4.2. Comparing Present Findings with Previous Research

The findings from the present study conducted in the Spanish cultural context seem to suggest that parenting styles without the warmth component are those related to the greatest delinquency during adolescence and the lower adjustment. Hence, it seems that parental warmth is an essential component of socialization in this cultural context (i.e., Europe), while strictness might be unnecessary to achieve effective socialization, in line with some recent studies, most of them with adolescents [12,24,48,61].

In addition, as opposed to classical research with middle-class European American families, the findings of this study revealed that parental strictness seems to be unnecessary when it is accompanied by parental warmth (indulgent and authoritative children reported the same scores) and even harmful when is not accompanied by parental warmth (children from authoritarian families had lower adjustment). The present study examined delinquency during adolescence, one of the most extreme forms of deviance against social standards usually examined in parenting studies [21,82]. On the one hand, contrary to the present findings, classical results from European American families identified that adolescents with lower rates of delinquency are those from families with strictness (i.e., authoritative and authoritarian) [21,28]. According to classical research, although adolescents from indulgent families benefit from parental warmth as much as those from authoritative families, they pay the cost for the lack of family control by reporting high delinquency rates, as do those from neglectful families [21,28]. In agreement with classical research, only parental strictness protects against deviance in its most drastic form (delinquency), but also in other forms such as drug use [21,28]. On the other hand, as opposed to results from European American families, the present findings seem to suggest that the use of reasoning and dialogue within an emotional climate of confidence and security (i.e., parenting styles characterized by warmth) is enough to transmit the social norms.

Among the objectives of socialization is not only to prevent social problems but also to favor the development of the individual as someone valuable and capable of doing well at school and work [9,10]. In this sense, the present results also identify that warmth is the key parental component that ensures maximum benefits for adolescent and adult children to feel valued and with good qualities (i.e., good self-esteem), to be confident in their abilities both at school and at work (i.e., good academic/professional self-concept), and to have as a priority the values of benevolence, which are those that favor empathic and prosocial behaviors.

### 4.3. The Optimal Parenting across the Globe within the Two-Dimensional Framework

Another contribution of the present study is that allows researchers across the world to examine parental socialization within a common theoretical framework, the so-called two-dimensional model [10], based on the two main parental dimensions (i.e., warmth and strictness) and the four parental styles that arise from the combination of the two dimensions (i.e., authoritative, authoritarian, indulgent and neglectful). The use of the same theoretical model makes it possible to compare the results of different studies carried out in different countries and cultural contexts.

The cultural context in which parental socialization takes place is key to understanding the discrepant results of the literature [13,41]. According to classical results, it was identified the benefits of strictness combined with warmth (i.e., the authoritative style). However, at least in this cultural context analyzed (i.e., European countries), it seems that the greatest adjustment is only associated with parental warmth without strictness (i.e., the indulgent style). Additionally, to test which is the best parenting in a particular context, it is quite important to use the same procedure (the use of a split procedure to assign families to the parenting groups) as well as the same research design and statistical analysis used in the previous studies in European American families [21,28,82] and other recent studies [24,132]. In this sense, the present research follows the same techniques, procedure, research design, and statistical analysis to test which parenting style is beneficial or harmful for adjustment in adolescents and adult children.

Addressing the debate regarding the best parenting style, the results do not support the universality of the authoritative style as the only optimal style and add more evidence that the context plays a key role in the effectiveness of parenting styles. Moreover, that the results advocate the implementation of educational programs based on warmth is consistent with other studies carried out in the Spanish context, or in Latin American or Southern European countries [13,44,60]. Thus, this study extends the evidence on the benefits of the indulgent parenting style, in the short and long term, and on four developmental criteria: academic/professional self-concept, self-esteem, delinquency during adolescence, and benevolence values.

### 4.4. Extending the Study of Parental Socialization beyond Adolescence

Another important contribution of the present study is that it provides empirical evidence regarding the relationship between parenting styles and psychological adjustment beyond adolescence—extending the importance of socialization when this process has been completed [3,4,62]. Adolescents, but also adult children, socialized by attachment-based families benefit from having both lower rates of adolescent delinquency and good adjustment. Specifically, according to the multivariate analysis, neither interaction effect of parenting styles by sex nor parenting styles by age were found for the adjustment criteria (self-esteem, self-concept, delinquency during adolescence, and benevolence values) suggesting that parental warmth without strictness (i.e., the indulgent style) is related to the same benefits in adjustment for sons and daughters as well as in adolescence (during parental socialization) and beyond (in adulthood and old age). In this line, the results seem to support the maintenance of the benefits of successful socialization over time [28,133,134].

Although it was not the focus of the research, some sex and age-related differences were found in line with some other studies [8,14,107]. Males showed higher self-esteem, lower academic/professional self-concept, especially in adolescence, lower expression of benevolence values, and higher rates of delinquency during adolescence, especially young adults. Additionally, in academic/professional self-concept the greatest scores were obtained by adolescents and the highest but middle-aged adults (young and older adults were in a middle position). In self-esteem, the lowest scores correspond to adolescents. In delinquency, young adults were those who had the greatest rates during their adolescence, followed by adolescents and middle-aged adults, while the lowest scores corresponded to older adults. Finally, in benevolence values, middle-aged adults showed greater scores than young adults and adolescents.

Differences in adjustment in adolescence, adulthood, and old life can be explained by different influences within the family (e.g., parental socialization) and outside the family (e.g., peers or media) [12,49,66]. Despite all these influences, in the present study, there was found a consistent pattern between parenting styles and children’s adjustment.

### 4.5. Limitations

When it comes to the limitations of the study, it is worth highlighting its cross-sectional nature and the non-experimental methodology, so it is not possible to establish causal relationships between the variables studied. However, the measure of socialization has been the same for both adolescent and adult children, as have the criteria for psychosocial development. It should be considered that in the present study, parental socialization is captured through the main two dimensions to conform to the four parenting styles (i.e., authoritative, authoritarian, indulgent, and neglectful), which are different parenting typologies of other parenting studies that have captured parental socialization through the clustering method as LPA (i.e., latent profile analysis) [135,136,137]. Additionally, it should be considered that responses in the present study are offered by children who seem to have more accurate responses compared to parents [138,139]. The study of parental socialization beyond adolescence requires us to use the same measures of parental socialization as for adolescent children, but with the statements in the past tense as it followed in the previous studies [14,65,120,121]. Overall, past-tense reports of general items (e.g., parental socialization) without specific details (e.g., dates or specific events) are usually considered acceptable, especially in community studies [140,141]. Specifically, empirical data support the factorial invariance of the two-dimensional model (i.e., warmth and strictness) across sex and age [14]. In any case, caution should be exercised because, in the case of adult children, especially middle-aged and older adults, it has been a long time since they were raised by their parents until now [14,120,121,142]. However, according to the main findings of the present study, even though adolescents and adult children grew up at different times, differences in their adjustment are equally related to parenting styles with the same pattern: only those from warm and non-strict families (i.e., the indulgent) reported the highest academic/professional self-concept, self-esteem, and benevolent values, and the lowest delinquency during adolescence. Future studies should move towards longitudinal follow-ups of children after socialization [143], although these projects are highly costly and do not usually provide very different results from cross-sectional ones, although they do allow more specific relationships to be established. Finally, it should be considered that all participants were from middle-class families, so results should be extended in future studies to other settings and SES (e.g., risk neighborhoods).

## 5. Conclusions

The present work examines the relationship between parenting across age groups and its impact on psychosocial adjustment. Most of the previous studies have been focused on the impact of parents on the child’s adjustment as the parental socialization process occurs [12,60] or based on the study of parenting practices [4,59] rather than on parenting styles. However, the present study assesses the relationship between parenting styles in which children were raised and children’s psychosocial adjustment during adolescence and beyond in a European country (i.e., Spain). Present findings revealed that parenting characterized by warmth (i.e., indulgent and authoritative styles) is related to higher psychological adjustment, as opposed to neglectful and authoritarian styles, which are associated with the poorest results. Specifically, children raised by indulgent families presented similar or higher psychosocial adjustment than those from authoritative homes. These results disagree with other studies conducted in different cultures that state that authoritative [21,28,31] or authoritarian [20,43] parenting are related to the greatest scores in child adjustment. However, the present findings agree with the most recent evidence about parenting conducted in Latin American or Southern European countries that highlights the importance of warmth without strictness (i.e., indulgent parenting) to achieve the best psychosocial adjustment of the child [13,44,60].

## Figures and Tables

**Table 1:** Distribution of participants by parenting style.

	Total	Neglectful	Indulgent	Authoritative	Authoritarian
Frequency	2125	442	625	453	605
Percentage	100	20.8	29.4	21.3	28.5
Warmth					
*M*	64.71	56.87	73.60	72.65	55.30
*SD*	11.31	9.18	4.36	4.19	9.65
Strictness					
*M*	34.63	28.32	28.28	39.85	41.91
*SD*	8.32	5.47	5.37	4.84	5.47

**Table 2:** Correlations between adjustment criteria, mean and standard deviation.

	1	2	3	4
1. Academic/Professional Self-Concept	1	0.363 **	−0.130 **	0.412 **
2. Self-esteem		1	0.007	0.202 **
3. Delinquency during adolescence			1	−0.132 **
4. Benevolence values				1
*M*	7.48	3.24	1.23	7.97
*SD*	1.49	0.47	0.28	1.13

** *p* < 0.01.

**Table 3:** MANOVA (4 ^a^ × 2 ^b^ × 4 ^c^) for academic/professional self-concept, self-esteem, delinquency during adolescence, and benevolence values.

	Ʌ	*F*	*df_between_*	*df_error_*	*p*
(A) Parenting styles ^a^	0.861	26.83	12.0	5529.9	<0.001
(B) Sex ^b^	0.913	49.85	4.0	2090.0	<0.001
(C) Age groups ^c^	0.876	23.58	12.0	5529.9	<0.001
A × B	0.990	1.73	12.0	5529.9	0.055
A × C	0.981	1.10	36.0	7833.9	0.317
B × C	0.963	6.63	12.0	5529.9	<0.001
A × B × C	0.985	0.88	36.0	7833.9	0.671

a_1_ neglectful, a_2_ indulgent, a_3_ authoritative, and a_4_ authoritarian. b_1_ women, y b_2_ men. c_1_ adolescents (12–18 years), c_2_ young adults (19–35 years), c_3_ middle-aged (36–59 years), and c_4_ older (60 years and older).

**Table 4:** Means, standard deviations, univariate *F*-values, and Bonferroni’s post hoc analysis of parenting styles for academic/professional self-concept, self-esteem, delinquency during adolescence, and benevolence values.

	Parenting Styles					
Adjustment Criteria	Neglectful	Indulgent	Authoritative	Authoritarian	*F*(3, 2093)	*p*
Academic/work self-concept	7.09 ^b^	7.90 ^a^	7.81 ^a^	7.10 ^b^	54.71	<0.001
	(1.49)	(1.24)	(1.38)	(1.64)		
Self-esteem	3.15 ^b^	3.35 ^a^	3.36 ^a^	3.09 ^b^	53.95	<0.001
	(0.45)	(0.45)	(0.41)	(0.47)		
Delinquency during adolescence	1.26 ^a^	1.20 ^b^	1.20 ^b^	1.27 ^a^	12.19	<0.001
	(0.30)	(0.24)	(0.25)	(0.33)		
Benevolence values	7.56 ^c^	8.22 ^a^	8.22 ^a^	7.81 ^b^	44.81	<0.001
	(1.21)	(1.03)	(0.97)	(1.16)		

Bonferroni test α = 0.05; a > b > c.

**Table 5:** Means, standard deviations, univariate *F*-values of sex for academic/professional self-concept, self-esteem, delinquency during adolescence, and benevolence values.

	*Sex*			
Adjustment Criteria	Women	Men	*F*(1, 2093)	*p*
Academic/work Self-concept	7.60	7.33	7.58	0.006
	(1.45)	(1.54)		
Self-esteem	3.20	3.30	53.95	<0.001
	(0.48)	(0.44)		
Delinquency during adolescence	1.17	1.32	150.84	<0.001
	(0.21)	(0.34)		
Benevolence values	8.07	7.82	24.84	<0.001
	(1.12)	(1.12)		

**Table 6:** Means, standard deviations, univariate *F*-values, and Bonferroni’s post hoc analysis of age group for academic/professional self-concept, self-esteem, delinquency during adolescence, and benevolence values.

	Age					
Adjustment Criteria	Adolescents	Young Adults	Middle-Aged Adults	Older Adults	*F*(3, 2093)	*p*
Academic/work Self-concept	7.05 ^c^	7.43 ^b^	8.10 ^a^	7.44 ^b^	49.15	<0.001
	(1.62)	(1.34)	(1.16)	(1.62)		
Self-esteem	3.15 ^b^	3.25 ^a^	3.31 ^a^	3.26 ^a^	11.02	<0.001
	(0.48)	(0.50)	(0.42)	(0.43)		
Delinquency during adolescence	1.22 ^b^	1.31 ^a^	1.23 ^b^	1.13 ^c^	41.45	<0.001
	(0.28)	(0.33)	(0.26)	(0.20)		
Benevolence values	7.78 ^b^	7.94 ^b^	8.19 ^a^	8.01	10.34	<0.001
	(1.18)	(1.08)	(1.06)	(1.15)		

Bonferroni test α = 0.05; a > b > c.

## Data Availability

Data can be made available for consultation upon request to the corresponding author and with permission of the participants of the study.
